# Editorial: Active components in psychotherapy: towards an integrative model of the mechanisms of therapeutic change

**DOI:** 10.3389/fpsyg.2023.1227477

**Published:** 2023-08-03

**Authors:** Rocío Guil, Antonio Romero-Moreno, Ricardo Tejeiro

**Affiliations:** ^1^Department of Psychology, University of Cádiz, Cádiz, Spain; ^2^School of Psychology, University of Liverpool, Liverpool, United Kingdom

**Keywords:** psychotherapeutic effectiveness, common factors, psychotherapy process research, therapist-patient bond, therapist's attributions

Psychotherapy outcomes have long been a subject of debate regarding the relative importance of specific techniques vs. common factors. Advocates for the former position contend that techniques with demonstrated efficacy play a crucial role in modifying dysfunctional thoughts, behaviors, and emotions, and emphasize the importance of evidence-based protocols and manualized treatments in addressing specific symptoms or disorders (Giles, 1993; Pérez Álvarez, 1996; Tolin, 2010; González-Blanch and Carral-Fernández, 2017). On the other end of the spectrum, a growing body of research emphasizes the significance of common factors – relational and contextual aspects shared across therapeutic modalities – in promoting positive outcomes (Lambert and Ogles, 2004; Karson and Fox, 2010; Wampold, 2017).

Key among these factors is the therapeutic alliance (Araya and Porter, 2017; Norcross and Wampold, 2019), characterized by trust, collaboration, and a positive working relationship between therapist and client, which creates a safe and supportive environment where clients feel heard, understood, and able to explore and change. Client factors – such as motivation, self-efficacy, and expectations – as well as therapist factors – empathy, positive regard, and cultural sensitivity – are important contributors to the therapeutic process (Romero-Moreno et al., 2021). Taken to the extreme, this view might imply that specific techniques or theories play a secondary role compared to these common factors (Wachtel et al., 2020).

In response to the debate, an integrative model of therapeutic change which posits that effective therapy results from the synergistic interaction between evidence-based techniques and the therapeutic relationship has gained prominence (Romero-Moreno, 2008; Caro, 2018). Common factors would create the context for change but, as del Río Olvera et al. summarize in their article in the present special issue, they “are perhaps considered necessary, but clearly insufficient alone.” Specific techniques would then be needed to provide the necessary framework and structure to address symptomatology and maladaptive patterns. This integrative approach emphasizes the importance of tailoring interventions to individual clients, considering their unique characteristics, needs, and preferences, and acknowledging that different clients may respond better to specific techniques or benefit more from the therapeutic alliance.

Further research is warranted to deepen our understanding of the complex mechanisms underlying therapeutic change. This Research Topic in Frontiers in Psychology represents a significant step in this direction by presenting valuable evidence from quantitative and qualitative research on a variety of samples from four different countries.

The article by del Río Olvera et al. from Universidad de Cadiz focuses on the first of the common factors mentioned above, the therapeutic alliance. Based on an exploratory quantitative study of 34 patients in a university psychological service, the authors show that the therapeutic alliance is forged fundamentally during the first session of the intervention, remaining relatively stable during the following ones even when the clients perceive that they are getting closer to their therapeutic goal. In addition to some promising methodological contributions (such as the use of the Dual SATIS method of analysis based on the follow-up of the measurements of each session), the article questions the commonly assumed reciprocal relationship between the therapeutic alliance and symptoms, in which symptoms predict the alliance and the alliance predicts the symptoms.

From a completely different approach, Fiskum et al. discuss the importance of developmental and transactional processes in relationships between children and their carers as a basis for intervention on internalizing difficulties. Through interviews with 13 parents of nine Norwegian children, they show that time-limited intersubjective child psychotherapy (TIC) improves children's ability to understand and regulate themselves, but also parenting skills and, fundamentally, mutual transactions between children and parents. The key take-away from this research may be that ICT is an effective integrative approach to therapy and that, as the authors conclude, “the therapeutic processes could come alive outside of the therapy room.”

In the third article, Mariani et al. present an instrument for measuring the free expression of thoughts that psychoanalysis proposes as a basis for therapy. To do this, they resort to the judgment of a large sample of experienced Italian psychoanalysts in two successive quantitative studies – factorial analysis and validity – that support the adequacy of the instrument.

The last of the articles in this special issue uses a case study to support the paradigm shift in professional counseling from the “person-job match” model toward a comprehensive intervention focused on personality traits, career adaptability and life issues. Its authors, Wang and Liu, demonstrate the effectiveness of Life Design Counseling (LDC) not only for the simple choice of subjects, but also in potentially psychotherapeutic aspects such as attention, self-awareness, self-determination, and behavior directed toward effective achievement of goals.

These four articles, varied in their focus, objectives, and methods, converge in the investigation not only of the efficacy of specific techniques, but also of their interaction with common factors. Our knowledge in this area is still very limited and further research effort is needed to develop evidence-based practices, refine psychotherapy interventions, and improve outcomes for people seeking psychological help.

## Author contributions

RG, AR-M, and RT: conception, literature review, and writing draft and revisions. All authors contributed to the article and approved the submitted version.
